# Novel Inhibitor Cystine Knot Peptides from *Momordica charantia*

**DOI:** 10.1371/journal.pone.0075334

**Published:** 2013-10-08

**Authors:** Wen-Jun He, Lai Yue Chan, Richard J. Clark, Jun Tang, Guang-Zhi Zeng, Octavio L. Franco, Cinzia Cantacessi, David J. Craik, Norelle L. Daly, Ning-Hua Tan

**Affiliations:** 1 State Key Laboratory of Phytochemistry and Plant Resources in West China, Kunming Institute of Botany, Chinese Academy of Sciences, Kunming, Yunnan, People’s Republic of China; 2 Institute for Molecular Bioscience, The University of Queensland, Brisbane, Queensland, Australia; 3 School of Biomedical Sciences, The University of Queensland, Brisbane, Queensland, Australia; 4 Centro de Análises Proteômicas e Bioquímicas, Programa de Pós-Graduação em Ciências Genômicas e Biotecnologia, Universidade Católica de Brasília, Brasília-DF, Brazil; 5 Centre for Biodiscovery and Molecular Development of Therapeutics, James Cook University, Cairns, Queensland, Australia; MRC National Institute for Medical Research, United Kingdom

## Abstract

Two new peptides, MCh-1 and MCh-2, along with three known trypsin inhibitors (MCTI-I, MCTI-II and MCTI-III), were isolated from the seeds of the tropical vine *Momordica charantia*. The sequences of the peptides were determined using mass spectrometry and NMR spectroscopy. Using a strategy involving partial reduction and stepwise alkylation of the peptides, followed by enzymatic digestion and tandem mass spectrometry sequencing, the disulfide connectivity of MCh-1 was elucidated to be CysI-CysIV, CysII-CysV and CysIII-CysVI. The three-dimensional structures of MCh-1 and MCh-2 were determined using NMR spectroscopy and found to contain the inhibitor cystine knot (ICK) motif. The sequences of the novel peptides differ significantly from peptides previously isolated from this plant. Therefore, this study expands the known peptide diversity in *M. charantia* and the range of sequences that can be accommodated by the ICK motif. Furthermore, we show that a stable two-disulfide intermediate is involved in the oxidative folding of MCh-1. This disulfide intermediate is structurally homologous to the proposed ancestral fold of ICK peptides, and provides a possible pathway for the evolution of this structural motif, which is highly prevalent in nature.

## Introduction

Small disulfide-rich peptides from plants and animals have diverse structures and bioactivities, and many have potential therapeutic applications [1]. The Cucurbitaceae plant family is a rich source of bioactive peptides with more than 60 disulfide-rich peptides isolated from over 10 species [2]. One species that has been of particular interest is *Momordica charantia* Linn., a tropical and subtropical vine, which is widely grown as a vegetable. It is commonly known as bitter gourd or bitter melon because the fruit is among the most bitter of all fruits. The roots, vines and seeds of *M. charantia* are used in traditional Chinese medicines [3]. Several serine protease inhibitors have been isolated and characterized from the seeds [2], [4]–[7]. These inhibitors are classified as squash trypsin inhibitors and are small (∼30 residue) disulfide-rich peptides containing three-disulfide bonds [2]. Members of this family share the characteristic feature of an inhibitor cystine knot (ICK) motif [8], [9], in which an embedded ring, formed by the CysI-CysIV, CysII-CysV disulfide bonds and their connecting peptide backbone segments, is penetrated by the CysIII-CysVI disulfide bond.

Major challenges in the study of disulfide-rich peptides include determination of their disulfide connectivity and synthesis of wild type and mutant peptides to explore structure-activity relationships [10], [11]. NMR is of significant value for the structural investigation of small disulfide-rich peptides, but a limitation of NMR is that it is difficult to unambiguously define the disulfide connectivity for cysteine-rich peptides [10], [12] due to the close packing of the cysteine residues. Therefore, the prior determination of disulfide connectivity is important in the NMR structure determination process. The traditional approach to assign the disulfide connectivity of peptides and proteins involves enzymatic digestion and disulfide mapping of the digestion fragments by mass spectrometry (MS) or N-terminal sequencing. This is generally not feasible for cystine-rich peptides because of the compact packing of the cysteine residues and resistance to enzymatic digestion. Approaches [10], [11], [13]–[16] involving partial reduction, stepwise alkylation, enzymatic digestion and MS were developed in the current study to overcome these problems [10], [17].

Characterization of the intermediates that transiently occur during oxidative refolding and reductive unfolding is necessary for a comprehensive understanding of the thermodynamic transition between folded and unfolded states, which in turn may lead to improved synthetic strategies [18], [19]. Characterizing folding intermediates is of significant challenge because they are not easily trapped [18], [20]. However, the relative stability of the intermediates of one of the peptides discovered in this study, MCh-1, enabled us to characterize the disulfide bonds present. Furthermore, the disulfide connectivities and folding pathways have great significance for our understanding of peptide structure, dynamics, stability, and ultimately function.

Recent studies suggest that we are only beginning to appreciate the significant diversity of bioactive disulfide-rich peptides from plants [21]–[27]. In the current study a chemical and biochemical investigation of the seeds of *M. charantia* was undertaken. This analysis led to the isolation and characterization of novel peptides that share no sequence homology with known peptides but adopt an ICK motif. MS data characterizing the intermediates from the partial reduction and oxidative refolding pathways demonstrated the disulfide linkage pattern in MCh-1 as CysI-CysIV, CysII-CysV and CysIII-CysVI. The new peptides were screened in several biological assays, including trypsin inhibition, antimalarial and cytotoxicity assays.

## Experimental Procedures

### General Experimental Procedures

Masses were analyzed on a Micromass LCT mass spectrometer equipped with an electrospray ionization source. For MALDI-TOF MS analysis, a Voyager DE-STR mass spectrometer (Applied Biosystems) was used and the data were collected between 300 and 4000 Da. Nanospray tandem mass spectrometry (MS/MS) experiments were conducted using the QStar spectrometer; the ion spray voltage was applied between 900 and 1100 V, and the data were acquired at *m/z* 200–2000 for ESI-TOF MS spectra and *m/z* 70–2000 for product ion spectra. The collision energy for peptide fragmentation was varied between 15 and 90 V. Amino acid composition analysis was conducted at the Howard Florey Institute, the University of Melbourne, Australia. Each peptide was hydrolyzed for 24 h with 6 N HCl at 110°C, and amino acids were analyzed using the Waters AccQ-Tag chemistry. Semipreparative RP-HPLC was performed on an Agilent 1100 apparatus equipped with a UV detector, ZORBAX Eclipse XDB C18 (Agilent, 9.4 mm×250 mm, 5 µm, 80 Å) and Phenomenex Jupiter C18 (10 mm×250 mm, 5 µm, 300 Å) columns at a flow rate of 3 mL/min, and a Phenomenex Jupiter C18 (4.6 mm×250 mm, 5 µm, 300 Å) column at a flow rate of 1 mL/min. Analytical RP-HPLC was performed using a Phenomenex Jupiter C18 (2 mm×150 mm, 5 µm, 300 Å) column at a flow rate of 0.3 mL/min (solvent A: Milli-Q water with 0.05% TFA; solvent B: 90% acetonitrile in Milli-Q water with 0.045% TFA). LCMS was carried out on an Agilent Series 1100 HPLC system that was connected to an ESI-TOF mass spectrometer at a flow rate of 0.3 or 1 mL/min (solvent 1: Milli-Q water with 0.1% formic acid; solvent 2∶90% acetonitrile in Milli-Q water with 0.1% formic acid).

Column chromatography was performed using D101 (Tianjin Haiguang Chemistry Company, Tianjin, China), Sephadex LH-20 and Sephadex G-25 (Pharmacia Fine Chemical Co., Ltd., Sweden) resins. Thin layer chromatography was carried out on precoated silica gel GF_254_ glass plates (Qingdao Marine Chemical, Inc., China). Spots were detected with ninhydrin and Coomassie brilliant blue G-250 [21]–[23].

### Plant Material

The seeds, stems, fruits and vines of *M. charantia* were purchased from Guangxi Academy of Agricultural Sciences, Guangxi province, China. The plant was identified by Prof. Ning-Hua Tan, Kunming Institute of Botany, Chinese Academy of Sciences. A voucher specimen (No. 0370356) was deposited at the herbarium of Kunming Institute of Botany, Chinese Academy of Sciences.

### Prescreen

Eighty grams each of the seeds (seed coats, decoated seeds), stems, fruits and vines was extracted first with acetone (5×1 L) and then with 50% aqueous ethanol (5×1 L) under reflux. The ethanol extract was concentrated and purified on a Sephadex LH-20 column (2 cm×80 cm) eluted with methanol.

### Extraction and Isolation of Peptides

The seeds of *M. charantia* (2.5 kg) were decoated, and the decoated, powdered seeds (1.4 kg) were extracted first with acetone (5×10 L) and then with 50% aqueous ethanol (5×10 L) under reflux. The ethanol extract was applied to a macroporous resin D101 column eluted with H_2_O and ethanol. The ethanol fraction (28 g) was applied to a Sephadex G-25 column (6 cm×60 cm) and eluted with 50 mM NH_4_HCO_3_ to give the peptide fraction. The peptides were further purified by semipreparative RP-HPLC to afford MCh-1 (130 mg), MCh-2 (235 mg), MCTI-I (260 mg), MCTI-II (83 mg), MCTI-III (275 mg) and MCTI-I(met-oxidized) (1 mg).

### Complete Reduction and Alkylation of Disulfide Bonds

The peptides (ca. 20 µg) were dissolved in 25 µL of buffer A (0.2 M Tris–HCl, pH 8.3) and 70 µL buffer B (8 M guanadinium–HCl) and freshly prepared 5 µL buffer C (160 mM dithiothreitol (DTT) in buffer A). Reduction of the disulfide bonds was done under nitrogen for 1 h at 37°C, and in the absence of light. Alkylation was performed using iodoacetamide (IAM). IAM (50 mg) was dissolved in Tris–HCl (250 µL) by heating at 65°C, and then added to the reduced peptides. After 1 min at room temperature, alkylation was terminated by adding 4 µL TFA.

### Sequence Determination of Peptides

To the fully reduced and alkylated peptides, 5 µL trypsin (40 µg/mL) or chymotrypsin (40 µg/mL) was added and the reaction allowed to proceed at 37°C for 3 h. The samples were desalted using Ziptips (Millipore) and stored at –20°C prior to analysis. The fragments resulting from the digestion were examined by MALDI-TOF MS followed by nanospray MS/MS analysis. The MS/MS data were examined and the peptides sequenced on the basis of the presence of both b- and y-series ions present (N- and C-terminal fragments).

### Selective Reduction and Stepwise Alkylation of Partially Reduced Disulfide Species

Selective reduction of the native MCh-1 (N) was done under nitrogen in 0.2 M citrate buffer at pH 3.5 and was optimized with respect to incubation time, incubation temperature and TCEP concentration to give the highest ratio of partially reduced disulfide species (the species with two disulfide bonds, IIa) relative to the fully reduced peptide (R). Selective reduction of MCh-1 was also done under nitrogen in 0.1 M ammonium bicarbonate (pH 8.5) in the presence or absence of 8 M guanadinium–HCl and was optimized with respect to incubation time, incubation temperature and TCEP or DTT concentration to give the highest ratio of partially reduced disulfide species relative to the fully reduced peptide. The optimal time, temperature and TCEP concentration for sampling were determined to be 8 min, 55°C, 0.1 M to give a similar percentage of partially reduced species compared with acidic conditions. Under the optimal acidic condition, large-scale selective reduction reactions were used to purify the major intermediates using semipreparative RP-HPLC at 1 mL/min. Fractions containing the IIa species were freeze-dried, resuspended in 0.2 M citrate buffer (pH 3.5), and then alkylated by adding *N*-ethylmaleimide (NEM, 0.06 M in 0.2 M citrate buffer, pH 3.5) in a volume equivalent to the concentrated fraction for 1 h. The alkylated peptides were then freeze-dried and dissolved in buffer A, in which the remaining disulfides were reduced using DTT and alkylated with IAM as described above.

### Oxidative Refolding of Fully Reduced MCh-1

The native MCh-1 was completely reduced and purified on semipreparative RP-HPLC at 1 or 3 mL/min, freeze-dried, and stored at –20°C for subsequent oxidative refolding studies.

Conditions for the oxidative refolding were optimized as follows: 50% isopropyl alcohol, 0.1 M ammonium bicarbonate (pH 8.5) either with 1 mM reduced glutathione (GSH) or not at room temperature. Aliquots were withdrawn at different time points, quenched with an equal volume of 4% aqueous TFA, and analyzed by RP-HPLC and LCMS. Samples were stored at –20°C.

### NMR Sample Analysis

Samples each of MCh-1 and MCh-2 were dissolved in 90% H_2_O/10% D_2_O. An additional 150 µL of CD_3_CN was added to the sample of MCh-1 to increase solubility. Spectra were recorded on a Bruker ARX 600 spectrometer at 290 K and 298 K. For resonance assignment a set of two-dimensional TOCSY [28] and NOESY [29] spectra with mixing times of 80 ms and 200 ms respectively, and a DQF-COSY [30] spectrum were recorded. All NMR spectra were processed using TOPSPIN (Bruker) and analyzed by Sparky [31].

The three-dimensional structures of MCh-1 and MCh-2 were calculated by deriving distance restraints from NOESY spectrum. Dihedral restraints were derived from ^3^ *J*_HNHα_ coupling constants measured from line shape analysis of antiphase cross-peak splitting in the DQF-COSY spectrum. A family of structures consistent with the experimental restraints was calculated using CYANA [32] and CNS [33]. A set of 100 structures was calculated, and the 20 lowest energy structures were selected and further analyzed using MolProbity [34]. Structures were analyzed using the programs PROCHECK [35] and PROMOTIF [36] to generate statistical analyses, including a Ramachandran analysis. The programs MolMol [37] and PyMol [38] were used to display the structural ensembles and surfaces of the peptides, respectively.

### Cytotoxicity Assay

The cytotoxicity of the peptides on breast cancer cells (MDA-MB-231) and non-cancerous human cells (HFF-1) was measured using a standard MTT (3-(4,5-dimethylthiazol-2-yl)-2,5-diphenyltetrazolium bromide, Sigma-Aldrich) assay. Cells were seeded in a 96-well flat-bottomed plate at a concentration of 5.0×10^4^ cells/cm^2^ in DMEM in 10% FBS, or DMEM in 15% FBS, and incubated at 37°C in an atmosphere of 5% CO_2_ in air. After 24 h, peptides were added in duplicate at the concentrations ranging from 0.1 µM to 100 µM to make up a final volume of 100 µL in each well and then incubated for 5 h. MTT solution (10 µL) in PBS (5 mg/mL) was added to each well and maintained for 1 h. Media was removed, and formazan crystals were resuspended in 100 µL of DMSO. Absorbance was then read at 600 nm [26]. Taxol was used as the positive control.

### Trypsin Inhibitory Assay

A stock solution of bovine pancreatic trypsin at 4.5 mg/mL in 1 mM HCl was prepared and stored on ice. Firstly, the trypsin stock was diluted to 0.45 mg/mL *in* 50 mM Tris/20 mM CaCl_2_ (pH 8.2) buffer. Next, the substrate was prepared by dissolving 0.435 mg/mL L-BAPNA (*N*α-benzoyl-L-arginine 4-nitroanilide hydrochloride, Sigma-Aldrich) in 1% DMSO/99% 50 mM Tris/20 mM CaCl_2_ buffer (pH 8.2). Four different peptide concentrations were tested starting from initial concentrations of 750 µM or 1.5 mM with 10-fold dilutions. The reaction mixture was prepared by dispensing 15 µL of 50 mM Tris/20 mM CaCl_2_ (pH 8.2) buffer, 5 µL of 0.45 mg/mL trypsin and followed by 5 µL of the tested peptide into each well of a 96-well plate in triplicate. A control without the tested peptide was used to measure 100% trypsin activity. Subsequently, 125 µL of 0.435 mg/mL substrate was added to the plate and incubated for 10 min at room temperature. The reaction was stopped by adding 25 µL of 30% acetic acid. Absorbance was measured at 410 nm using a Powerwave XS plate reader (Bio-Tek). Trypsin inhibition was calculated from the absorbance in the presence of the tested peptide as a percent of absorbance of the uninhibited trypsin [39]. MCoTI-II was used as the positive control [40].

### Antimalarial Assay

*Plasmodium falciparum* (FCR-3 strain) was maintained *in vitro* at 37°C in GIT medium containing human red blood cells (RBCs, type A) at 5% hematocrit in 24-well plates which were put in a CO_2_ incubator (5% CO_2_, 5% O_2_ and 90% N_2_) at 37°C [41]. Peptides were added in duplicate in distilled water or 0.25 M phosphoric acid and prepared in various concentrations. 10 µL of this solution was added to individual cells of the plates. Erythrocytes with 0.3% parasitemia were added to the cells of the above plates containing 990 µL of culture medium. The plates were incubated at 37°C for 72 h in the CO_2_ incubator. To test the antimalarial activity of peptides, thin smears were prepared from each culture and stained with Giemsa. Erythrocytes were examined under microscopy. Artemisinin was used as the positive control. Drug-free control cultures were assayed simultaneously [42], [43].

## Results

### Isolation and Characterization of Peptides

**Figure 1:**
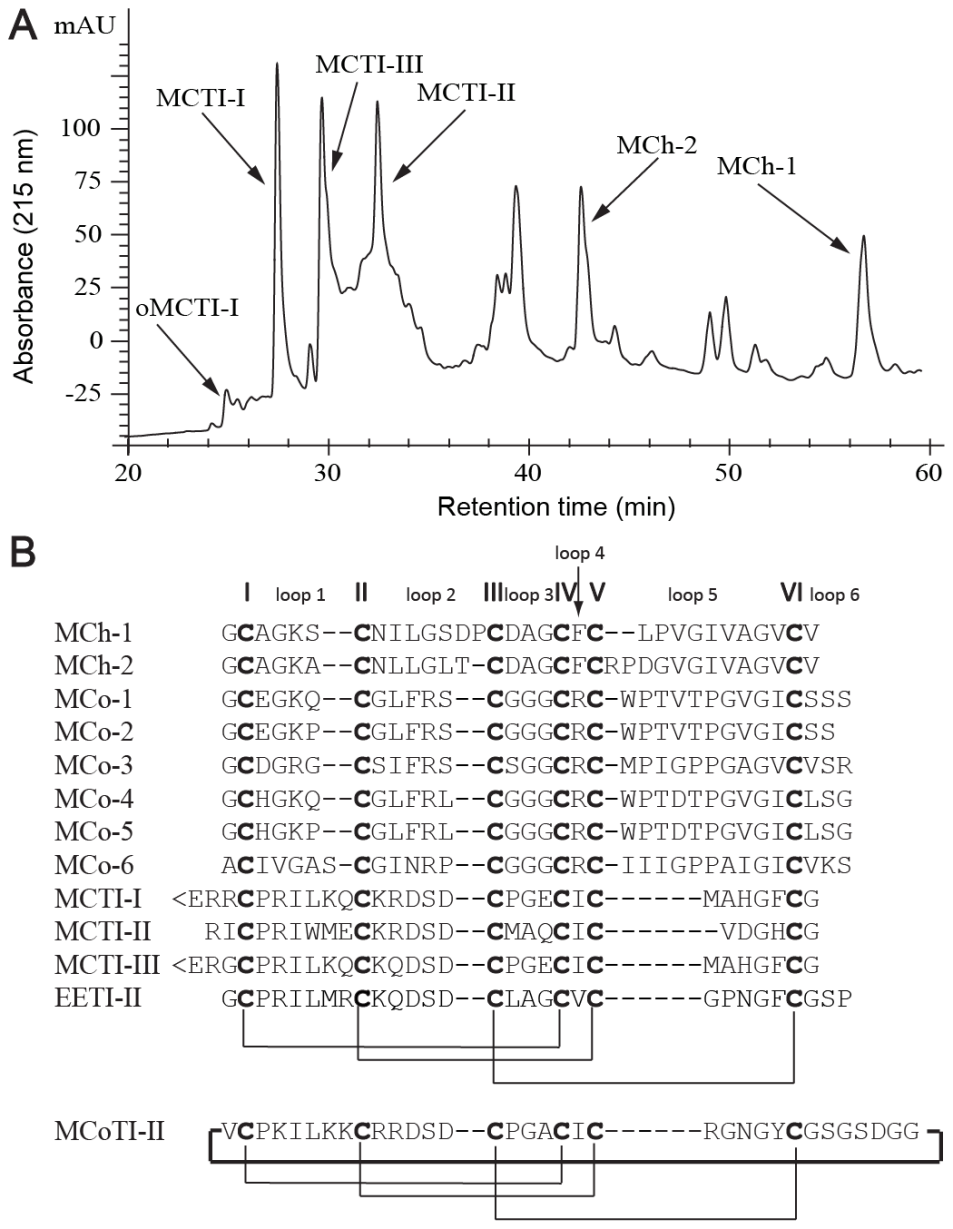
Isolation of novel peptides (MCh-1 and MCh-2) from *M. charantia* and sequence comparison with related peptides. (A) RP-HPLC trace of the crude peptide extract from *M. charantia* seeds showing the retention times and relative intensities of the different peptides. The gradient was 1% solvent B linear for 70 min at 3 mL/min. (B) Alignment of the sequences of MCh-1, MCh-2, MCo-1 to MCo-6 [27], [39], MCTI-I [4], MCTI-II [4], MCTI-III [5], EETI-II [19] and MCoTI-II [40]. Cysteine residues (numbered I–VI) are highlighted in bold and the backbone loops are numbered 1–6.

To gain a better understanding of the structural diversity of peptides from *M*. *charantia* we screened the seed coats, decoated seeds, stems, fruits and vines for the presence of peptides. Extracts were initially size-fractionated using a Sephadex LH-20 column. Fractions were collected and analyzed using a thin layer chromatography chemical method established for the isolation of peptides with similar properties [22], [23]. The peptide-containing fractions that eluted early from the extract of the decoated seeds were further examined by ESI-MS over a mass range corresponding to typical plant-derived disulfide-rich peptides (2500–4000 Da). Large-scale purification of the peptide-containing fractions from the 50% aqueous ethanol extract of the decoated seeds provided two new peptides, MCh-1 and MCh-2, as well as an oxidized methionine product, MCTI-I(met-oxidized), along with three known peptides (MCTI-I, MCTI-II and MCTI-III) [4], [5], as described in the Experimental Procedures. The separation profile obtained by RP-HPLC is shown in Figure 1A. The sequences of the peptides (Figure 1B) were determined by a combination of enzymatic digests, tandem MS sequencing, amino acid analysis and NMR.

The molecular mass of MCh-1 was determined by LCMS to be 3022 Da. Reduced, or reduced and alkylated, derivatives of this peptide gained 6 or 348 mass units respectively relative to the native peptide, as confirmed by MALDI-TOF MS and ESI-MS and shown in Table S1 in File S1. The mass increase after reduction or alkylation suggested that MCh-1 contained six cysteine residues involved in three disulfide bonds. The reduced peptide was digested with chymotrypsin and the resulting major fragments had monoisotopic masses of 1916.94 and 1128.52 Da. These fragments were sequenced using nanospray MS/MS and corresponded to the partial sequences GCAGKSCNILGSDPCDAGCF and CLPVGIVAGVCV (Table S1 in File S1). A combination of these fragments defined the complete sequence of MCh-1. Its sequence was confirmed with alkylation of the peptide with IAM, followed first by chymotrypsin or trypsin digestions and then MS/MS sequencing. The number and position of the Ile and Leu residues in the sequence of MCh-1 were determined by analysis of amino acid composition and NMR spectroscopy.

A similar approach was used to sequence and characterize MCh-2. The details on the sequence characterization are provided in Table S1 in the File S1.

### Determination of the Disulfide Connectivity of MCh-1

**Figure 2:**
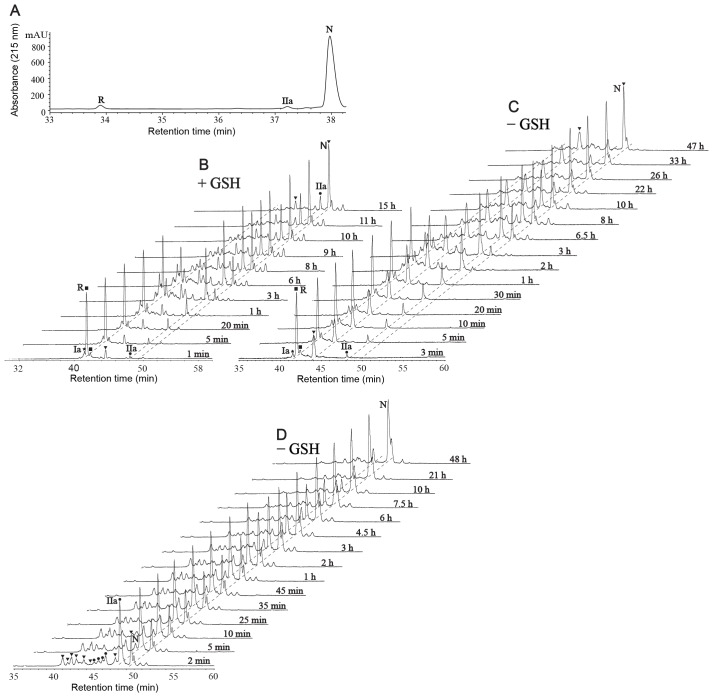
Reductive unfolding and oxidative refolding of MCh-1. (A) RP-HPLC trace of the selective reduction of MCh-1 using 0.1 M TCEP in 0.2 M citrate buffer at pH 3.5 at 65°C under nitrogen after 3 min. The elution positions of the fully reduced (R), the species with two disulfide bonds (IIa) and the native MCh-1 (N) are indicated. The gradient was 5% solvent B for 5 min, 5–80% solvent B linear at 0.3 mL/min in 40 min. (B & C) LCMS profiles of the refolding of the reduced MCh-1. Oxidative refolding was performed in 50% isopropyl alcohol, 0.1 M ammonium bicarbonate (pH 8.5) either with 1 mM GSH or not at room temperature. An equal volume of aliquots was withdrawn at different time points, quenched with an equal volume of 4% aqueous TFA, and analyzed by RP-HPLC and LCMS. The samples were stored at –20°C. “R” and “N” indicate the elution positions of the fully reduced and native MCh-1. “Ia” and “IIa” denote the partially reduced intermediates containing one (CysIII-CysVI) and two (CysII-CysV and CysIII-CysVI) disulfide bonds respectively. The gradient was 5% solvent 2 for 5 min, 5–35% solvent 2 linear at 0.3 mL/min in 60 min. (D) LCMS profiles of the refolding of IIa (Experimental conditions are same as C). “▾, •, ★, and ▪” indicate the three-disulfide, two-disulfide, and one-disulfide scrambled isomers, and fully reduced MCh-1, respectively.

**Figure 3:**
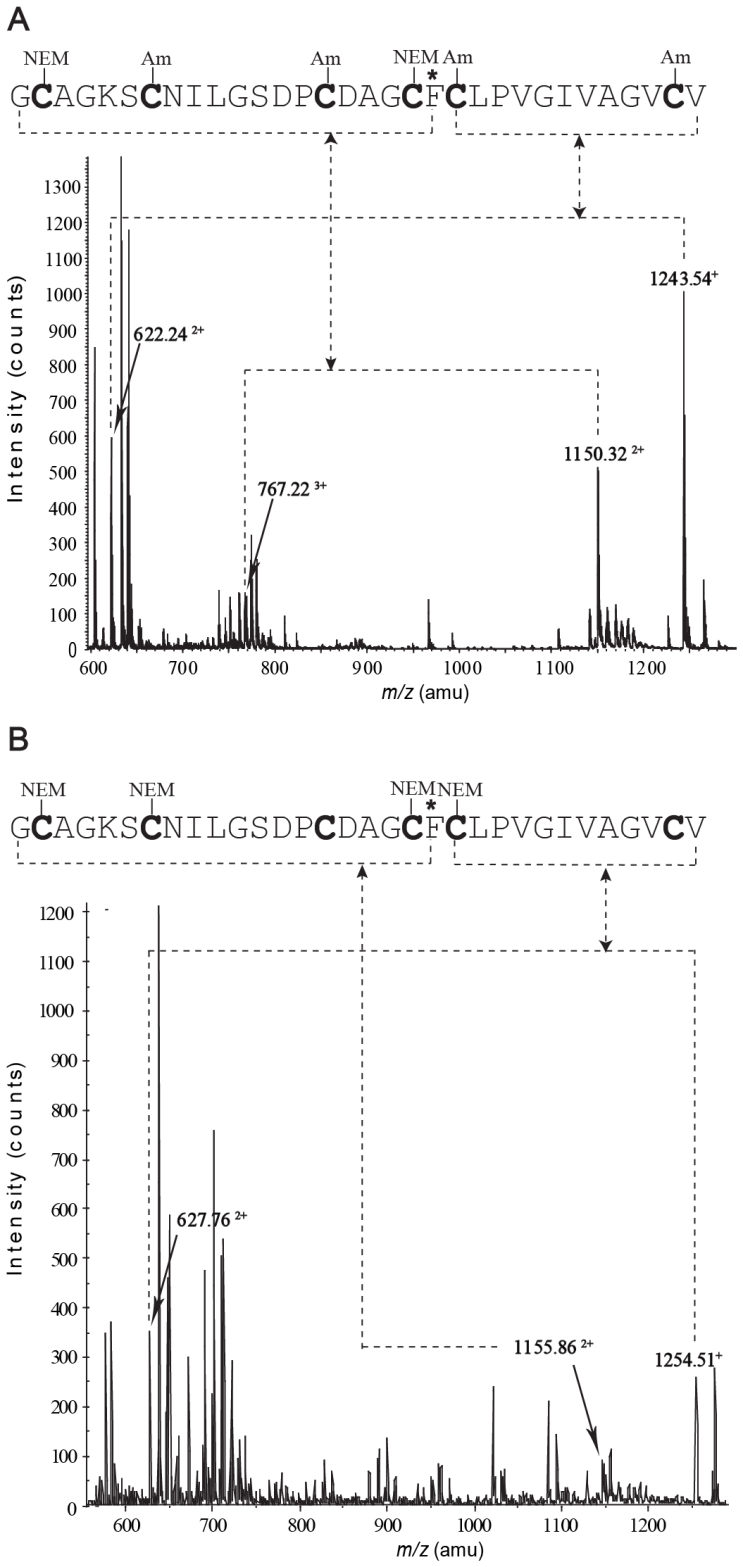
MS/MS identification of the disulfide connectivity for MCh-1. (A) ESI-TOF MS spectrum of the chymotrypsin digest of alkylated IIa isolated from the selective reduction experiments of the native MCh-1. (B) ESI-TOF MS spectrum of chymotrypsin digest of alkylated Ia isolated from the oxidative refolding of the fully reduced MCh-1. The phenylalanine residues that define the digestion positions are indicated with asterisks.

**Figure 4:**
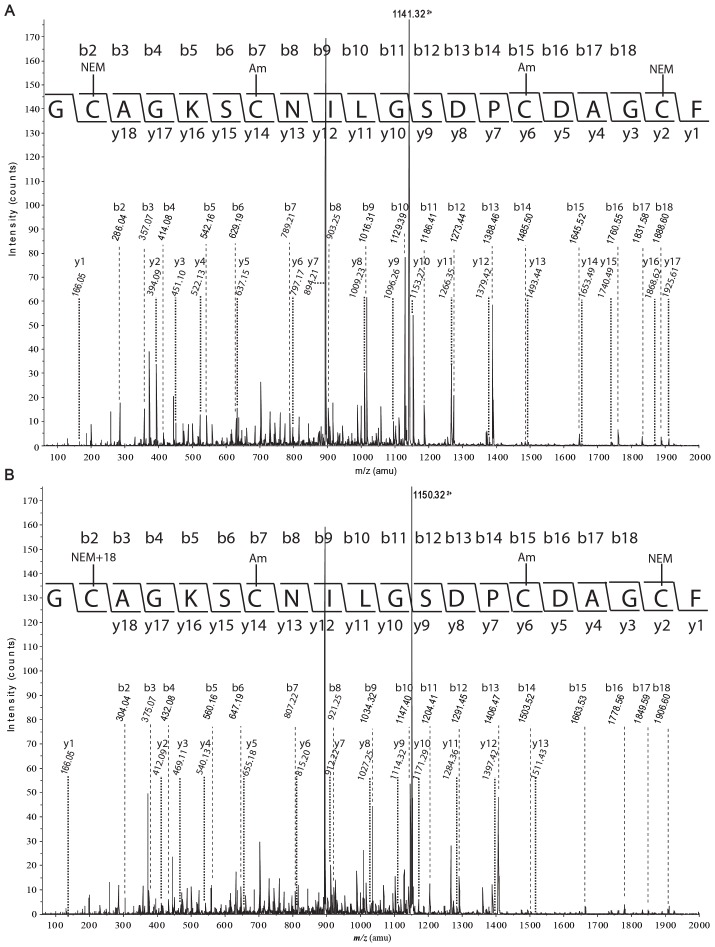
MS/MS spectra of (A) the doubly charged ion *m/z* 1141.32 and (B) its +18 adduct ion *m/z* 1150.32 from the digestion of the alkylated IIa. The partial sequence of the peptide, alkylation type and position, the detected b- and y- series of ions are shown. “b and y” designate ions having the charge retained on the N-terminal fragment and the C-terminal fragment, respectively.

A selective reduction approach [10], [11] was used to determine the disulfide connectivity of MCh-1. The optimal time and temperature for the observation of intermediates during reduction was 3 min at 65°C (Figure 2A), when the reduction was carried out under nitrogen in 0.2 M citrate buffer at pH 3.5. Acidic conditions were used to avoid rearrangement of the disulfide bonds [44], [45]. However, similar results were obtained when the reduction was done at pH 9.5. A large-scale partial reduction was carried out at pH 3.5 and allowed the isolation of a major intermediate, IIa. This intermediate was alkylated with NEM, purified by RP-HPLC and the molecular weight of the NEM-alkylated MCh-1 was determined to be 3273.08 Da by LCMS and MALDI-TOF MS, corresponding to the product containing two NEM groups. The NEM-alkylated MCh-1 was fully reduced and modified with IAM. The resulting peptide was isolated and the molecular mass of the alkylated peptide was determined to be 3505.28 Da by LCMS and MALDI-TOF MS, consistent with alkylation of the four remaining cysteine residues with IAM. The NEM- and carboxamidomethyl (Am)-alkylated peptide was further treated with trypsin and chymotrypsin. As shown in Figure 3A, Figure 4 and Table S1 in File S1, analysis of the ESI-TOF MS spectrum of the chymotrypsin digest of the alkylated peptide indicated that CysI and CysIV were alkylated with NEM, and consequently involved in a disulfide bond in the native peptide. MS/MS sequencing of the fragments from the trypsin digestion, shown in Table S1 in File S1, confirmed this result.

The intermediate IIa converts to the fully reduced MCh-1 without significant buildup of a one-disulfide intermediate along the unfolding pathway (Figure 2A). However, a one-disulfide intermediate Ia was observed during the oxidative refolding see below, along with a two-disulfide intermediate (Figure 2B). Quantification of different species occurred during reductive unfolding and oxidative refolding in different time courses are shown in Figure S1 in File S1. Alkylation of the two-disulfide intermediate confirmed it was identical to the intermediate IIa observed during the reductive unfolding. Alkylation of the one-disulfide intermediate, Ia, with NEM resulted in a peptide with a mass of 3525.16 Da, corresponding to addition of four NEM groups. NEM-Alkylated Ia was subjected to complete reduction, enzymatic digestion and sequencing (Figure 3B and Table S1 in File S1), which indicated that the CysIII-CysVI disulfide bond was intact, and CysI, CysII, CysIV and CysV were alkylated with NEM. Combining the information gained from IIa and Ia suggests that the disulfide connectivity of MCh-1 is CysI-CysIV, CysII-CysV and CysIII-CysVI.

### Analysis of the Kinetics of the Oxidative Refolding of MCh-1

In addition to providing data on the disulfide connectivity, analysis of the intermediates of MCh-1 provided important insights into the oxidative folding pathway. Fully reduced MCh-1 can be refolded into the native conformation under basic conditions with or without GSH. However, the complexity of the intermediates present appears to increase in the presence of GSH. The oxidative refolding reaction was monitored by removing aliquots at selected time points and analyzing them using RP-HPLC and LC-MS (Figures 2B & 2C). Interestingly, the reduced peptide eluted before the native peptide on RP-HPLC. This behavior is similar to cyclotides, circular mini-proteins of 28–37 amino acid residues present in plants, which have a head-to-tail cyclic peptide backbone and an ICK motif [46]. The late elution of the native peptide presumably occurs because the interior of the molecule is occupied by the cysteine residues, thus forcing hydrophobic amino acids to be surface-exposed.

The folding pathway of MCh-1 was characterized by structural and kinetic analysis of acid-trapped folding intermediates. The most striking feature of the folding kinetics of MCh-1 is the rapid formation of the predominant intermediate IIa and the native peptide. Accumulation of the two-disulfide species occurs in both the selective reduction and the oxidative refolding processes; this suggests that it adopts a highly stable structure and represents a major kinetic trap during MCh-1 folding. The amounts of Ia and IIa increase during the early phase of the oxidative folding process, but decrease during later phases. The native form accumulates along the pathway of oxidative refolding, indicating that the native form is the most stable form.

The intermediate IIa was isolated and the folding reaction monitored with HPLC. Four minor two-disulfide scrambled isomers and six minor three-disulfide scrambled isomers were observed along the course of folding of IIa to form the native peptide as shown in Figure 2D. Under the experimental conditions used (see Experimental Procedures (50% isopropyl alcohol, 0.1 M ammonium bicarbonate (pH 8.5) at room temperature), the disulfide isomers are expected to be freely reversible, allowing the possible disulfide-bonded isomers to accumulate according to their relative stabilities. The native form eluted latest among the three-disulfide scrambled isomers along the oxidative folding pathway, consistent with it being the most stable form.

### Structural Analysis of MCh-1 and MCh-2 using NMR

**Figure 5:**
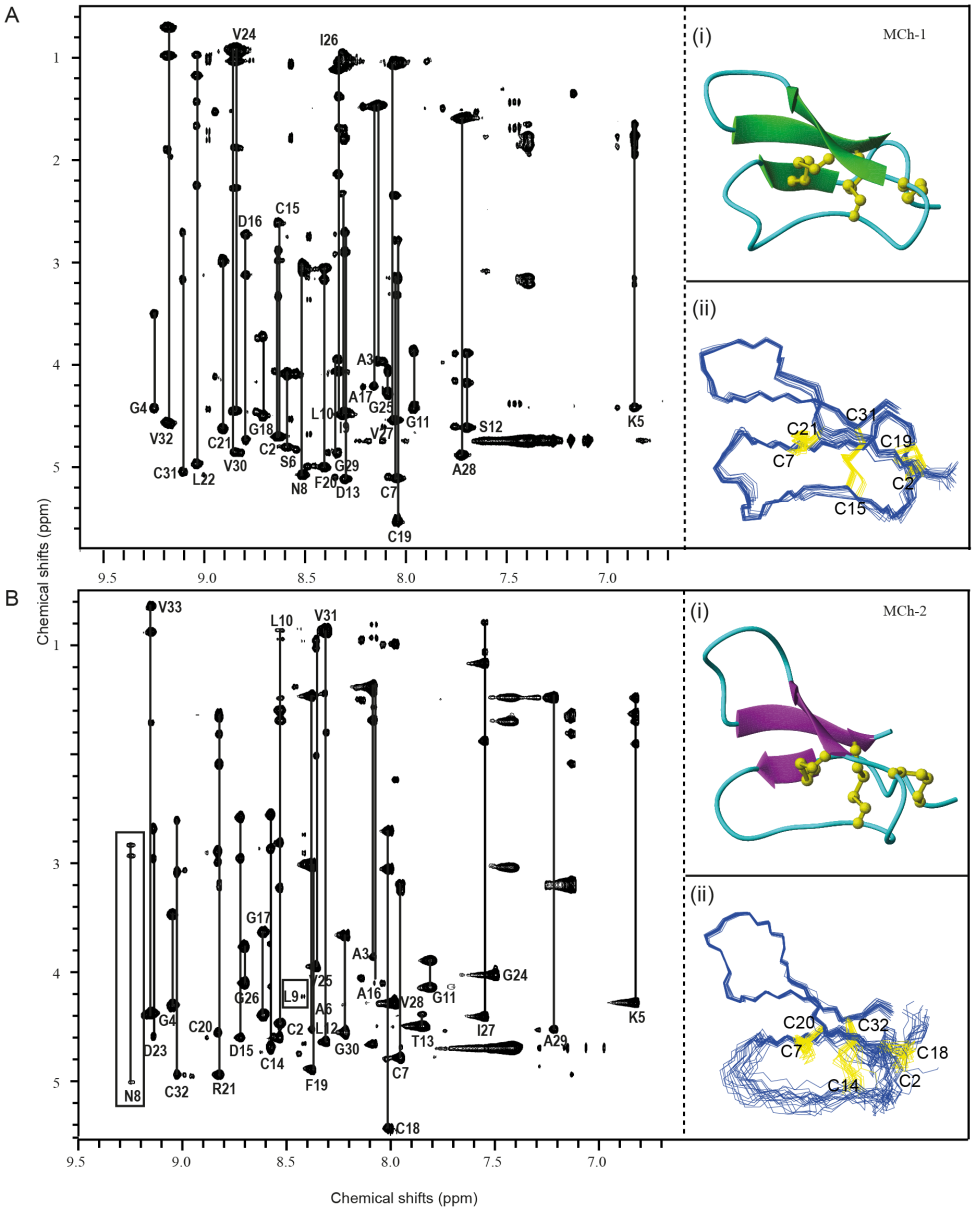
Structures of MCh-1 and MCh-2. (A) The TOCSY spectrum of MCh-1 along with a ribbon structural representation (i) and 20 lowest energy structures (ii). (B) The TOCSY spectrum of MCh-2 along with a ribbon structural representation (i) and 20 lowest energy structures (ii). The square brackets highlight the residues with broadened peaks resulting from flexibility.

The pattern of backbone resonance chemical shifts revealed in the TOCSY spectrum of MCh-1 (Figure S2A in File S1 and Figure 5) is consistent with that of a β-sheet structure on the basis of the well-dispersed amide peaks and the downfield-shifted α-proton signals. Sequential connectivities between neighboring spin systems were obtained from an analysis of H_α_-H_N+1_, H_N_-H_N+1_ and H_β_-H_N+1_ cross-peaks in the NOESY spectrum according to established procedures [47]. Further information on the NOESY fingerprint regions that show the connectivity between the αH of one residue to the NH proton of the sequential residue (H_α_-H_N+1_) of MCh-1 is shown in Figure S2B in File S1.

Three-dimensional structures of MCh-1 and MCh-2 were determined using 417 and 319 distance restraints; 13 and 19 dihedral angle restraints, respectively, derived from the NMR structural data (Table S2 in File S1). According to the reductive unfolding and oxidative refolding data, the disulfide bond connectivity is consistent with cystine knotted peptides (i.e., CysI-CysIV, CysII-CysV and CysIII-CysVI). Therefore, the MCh-1 and MCh-2 structures were determined on the basis of this known disulfide connectivity.

Structures of MCh-1 and MCh-2 were determined using structure calculation programs such as CYANA [32] and CNS [33]. The 20 lowest energy structures were chosen from 100 calculated structures. Most residues were observed in favored regions of the Ramachandran plot. The secondary structures of MCh-1 and MCh-2 contain an anti-parallel β-sheet, which is similar to MCo-1, a cystine knot peptide isolated from *M. cochinchinensis* [39]. Analysis of β-strands and β-turns positions in MCh-1 and MCh-2 sequences was carried out using PROMOTIF [36]. The three β-strands of MCh-1 and MCh-2 were from Lys5-Ile9, Phe20-Val24, Val27-Val32 and Cys7-Leu9, Phe19-Asp23, Val28-Val33, respectively (Figure 5). Furthermore, different types of β-turns were observed in MCh-1 and MCh-2. In MCh-1, type I (Asn8-Leu10), type II (Cys2-Lys5), and type IV (Ser12-Pro14, Asp16-Cys19, and Gly25-Ala28) β-turns were observed. In MCh-2 type II (Gly24-Ile27) and type IV (Ala3-Ala6, Asn8-Gly11, and Asp15-Cys18) β-turns are present. PROCHECK [35] was used to generate a Ramachandran plot and MolMol [37] was used to display structural representations. From the structural analysis, MCh-2 has more disorder in loop 2 than MCh-1. Several residues in loop 2 in MCh-2 have broadened peaks indicative of structural flexibility (Figure 5B). Structural data for MCh-1 and MCh-2 have been deposited in the Protein Data Bank (http://www.pdb.org) and the BMRB (http://www.bmrmb.wisc.edu). The PDB codes for MCh-1 and MCh-2 are 2M2Q and 2M2R, respectively and the BMRB code for both peptides is 18926.

### Biological Activities of MCh-1 and MCh-2

MCh-1 and MCh-2 were evaluated for trypsin inhibitory activity, antimalarial activity against *P. falciparum* (FCR-3 strain), and cytotoxic activity against MDA-MB-231, a human breast cancer cell line, and HFF-1, a non-cancerous human cell line. However, the peptides were not active in these assays.

## Discussion

The seeds of Cucurbitaceae species are emerging as a rich source of novel disulfide-rich peptides. In this study we isolated two peptides (MCh-1 and MCh-2) from the seeds of *M. charantia* that contain novel sequences and ICK structural motifs. Analysis of the oxidative refolding highlighted a common intermediate present in the folding of a variety of ICK peptides, despite variations in inter-cysteine loop sizes. This two-disulfide intermediate is surprisingly stable and provides new insights into how the cystine knot might have evolved from a simple disulfide framework.

The ICK is a structural motif present in a wide range of peptides and proteins isolated from insects, plants and animals [1], [48]. We used selective reduction, oxidative refolding, stepwise alkylation, and MS analysis to determine that the disulfide connectivity of MCh-1 is CysI-CysIV, CysII-CysV and CysIII-CysVI. Calculation of the three-dimensional structures of MCh-1 and MCh-2, using the derived connectivity as restraints, indicates that they contain the ICK motif, and can be added to the growing number of peptides in this structural family.

A comparison of the sequences of MCh-1 and MCh-2 with peptides isolated from the related species *M. cochinchinensis* shows that although the peptides all contain six cysteine residues, and loops 3 and 4 all contain the same number of residues, the other inter-cysteine loops are variable. Similarly, the sequences differ from the squash trypsin inhibitors isolated from *Momordica* species. These sequence differences are reflected in the different retention times observed on RP-HPLC, with the squash trypsin inhibitors being more hydrophilic than MCh-1 and MCh-2. The peptides also differ in activity, as MCh-1 and MCh-2 are not trypsin inhibitors. Given the differences in sequence and activity it is apparent these novel peptides belong to a new subfamily of ICK members.

**Figure 6:**
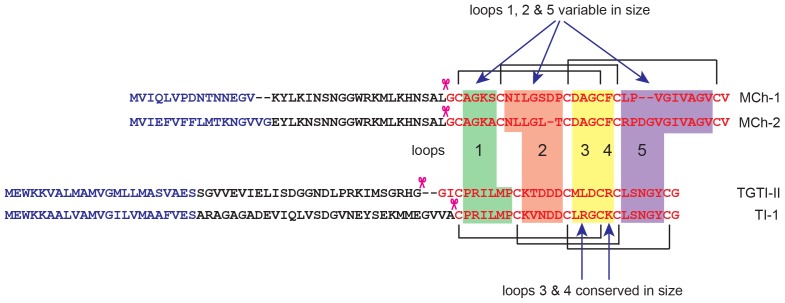
Predicted precursor proteins of MCh-1 and MCh-2 compared with the precursor proteins of TGTI-II [**53**] **and the** ***Trichosanthes*** **trypsin inhibitor (TI-1)** [**52**] **.** The latter two sequences were retranslated based in the Mylne et al. study [51]. The signal peptides are shown in blue and were predicted on the basis of SignalP3.0 [50], except for MCh-1, which was based on sequence similarity with MCh-2. The N-terminal region in the MCh-1 precursor is shorter than in MCh-2 and SignalP3.0 did not predict a signal peptide. Variations in loop sizes were also noted when MCh-1 and MCh-2 were compared to TGTI-II and TI-1. Loops 1, 2 and 5 were observed to vary in sizes except loops 3 and 4. Cleavage sites which yielded mature peptides (highlighted in red text) were indicated with pink scissors icons.

The peptide sequences of both MCh-1 and MCh-2 could be predicted from recent high-throughput transcriptomic data from *M. charantia* seeds [49] (Contigs MomordicaCtg_2933 and MomordicaCtg_57, respectively; cf. http://genomics.msu.edu/JO/blast/blast.html). The peptides appear to be encoded as small precursor proteins comprising a signal sequence, and a short pro-region followed by the mature domain, as shown in Figure 6. The signal peptide for MCh-2 was predicted from SignalP3.0 [50].

The gene sequences of several other peptides, predicted to contain ICK motifs, and present in the Cucurbitaceae family, have been determined recently [51]. These precursor proteins have similar architectures to MCh-1 and MCh-2, with the mature peptides at the terminal region of the precursor proteins. However, the cleavage sites that yield the mature peptide vary for these different disulfide-rich peptides. Known cleavage sites include after an alanine residue for a trypsin inhibitor TI-I from *Trichosanthes kirilowii* [52] and cleavage after a glycine residue to yield TGTI-II from the towel gourd [53], as shown in Figure 6. Both MCh-1 and MCh-2 appear to require cleavage after a leucine residue to yield the mature peptides. The diversity in the cleavage sites suggests that a range of proteases are involved in the maturation of plant ICK peptides.

The oxidative folding of MCh-1 was analyzed using RP-HPLC and MS. MCh-1 represents an ideal peptide for investigating the oxidative refolding process of the ICK motif since its *in vitro* oxidation is slow enough to allow the isolation and characterization of intermediates formed during folding. Although IIa was the major intermediate, numerous other intermediates were present in the oxidative refolding of MCh-1, both in the presence and in the absence of the shuffling reagent glutathione. Refolding of purified IIa resulted in numerous species, including two-disulfide and three-disulfide isomers. This complexity in the folding pathway indicates that IIa does not convert directly to the native form. By contrast with the oxidative refolding process, the reductive unfolding is very simple and IIa was the only intermediate observed.

**Figure 7:**
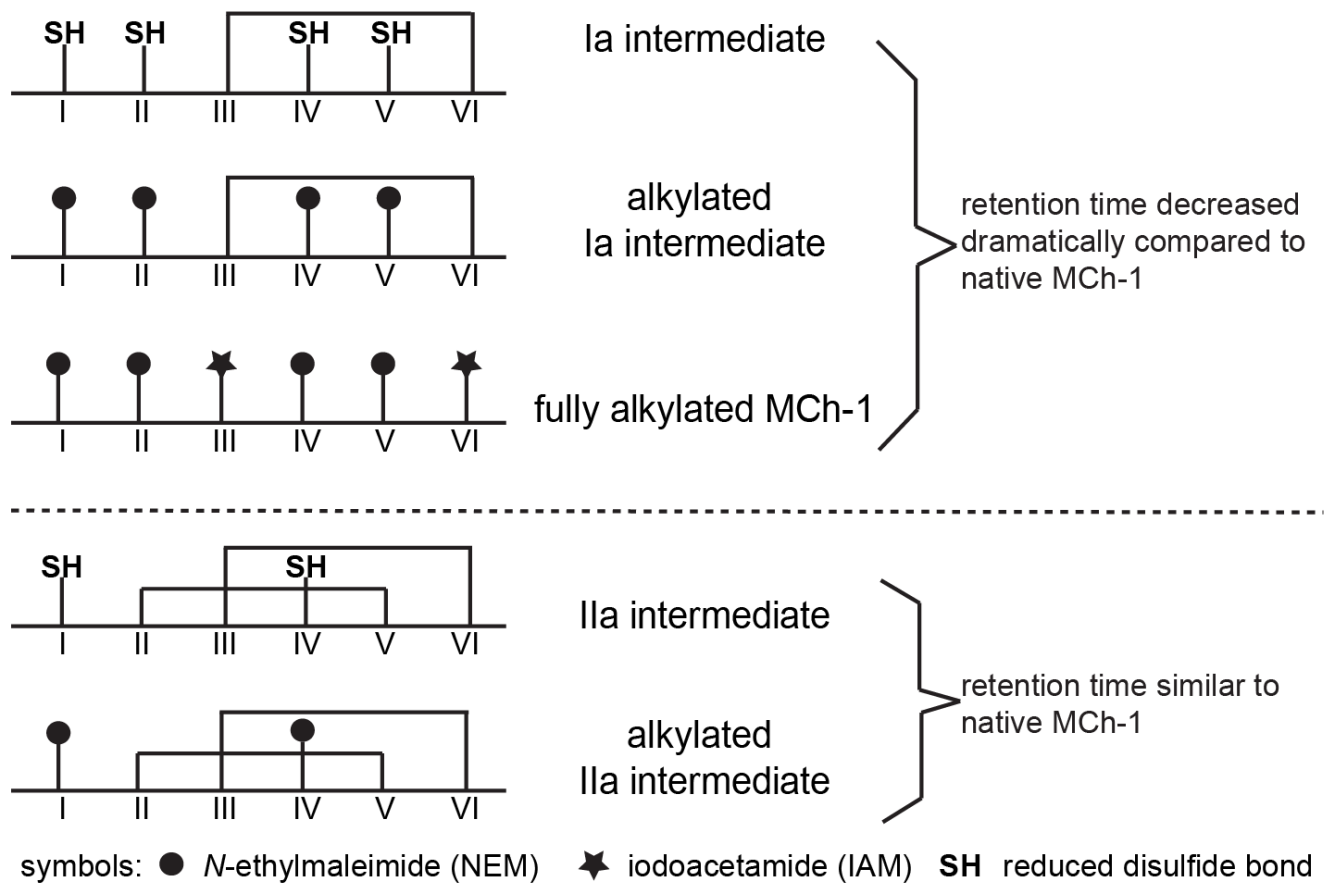
This schematic diagram represents the different folds of MCh-1 intermediates.

It is interesting to note the differences in retention times of the intermediates during the selective reduction and the stepwise alkylation, or the oxidative refolding and the stepwise alkylation. The intermediate IIa eluted just before the native peptide and the similarity of the retention times of the intermediate with that of the native peptide indicates that the three-dimensional structures are similar and that the intermediate also contains a compact structure. The retention time of NEM-alkylated IIa was almost the same as that of IIa. This similarity in retention times suggests that the NEM replacement did not affect the conformation of IIa. By contrast, the retention times of NEM-alkylated Ia, the fully alkylated MCh-1 and intermediate Ia decreased dramatically relative to the native peptide, which indicates that those structures are not compact. Different folds of MCh-1 intermediates are shown in Figure 7.

The two-disulfide intermediate observed during the folding of MCh-1 is equivalent to the major intermediates previously reported for the plant ICK peptides EETI-II [19], MCoTI-II [18] and kalata B1 [54]. The structures of these intermediates have been analyzed using NMR spectroscopy and shown to contain the native fold but lack the CysI-CysIV disulfide bond. EETI-II and MCoTI-II are both squash trypsin inhibitors with similar sequences, and do not share sequence similarity with kalata B1, with the exception of the six cysteine residues. However, both MCoTI-II and kalata B1 contain a cyclic backbone in addition to the ICK motif. Therefore this intermediate accumulates during folding in both cyclic and acyclic peptides and does not appear to have stringent sequence requirements given the diversity of sequences across these different peptides. Despite the conservation of the intermediate IIa in the folding of various ICK peptides, the pathways involved in the formation of the native peptide vary. The intermediate IIa observed during the folding of MCoTI-II appears to be the direct precursor to the native peptide [18], in contrast to the intermediate observed during the folding of kalata B1, which requires rearrangement of the disulfide bonds to form the native peptide [54]. The MCh-1 IIa intermediate is also likely to require rearrangement of the disulfide bonds based on the additional intermediates observed in the analysis of the folding of purified intermediate IIa.

The conservation of the two-disulfide intermediate implies an integral role in the folding of the ICK motif. Indeed, it is tempting to speculate that this intermediate is involved with the evolution of the ICK given it is equivalent to the proposed ancestral fold [55]. The disulfide-directed β-hairpin (DDH) comprising two-disulfide bonds equivalent to the CysII-CysV and CysIII-CysVI bonds in the ICK motif has been proposed to be the ancestral fold of the ICK [55]. This hypothesis has recently been supported by the discovery of a scorpion toxin containing the DDH motif. Based on this discovery it has been suggested that the derivation of the ICK motif into scorpion venoms is a result of a simple modification of the DDH fold. This scenario could equally be applied to the evolution of ICK peptides in plants, and the presence of the stable two-disulfide intermediate accumulating in the folding of diverse cystine-knot peptides might have facilitated this simple modification.

In conclusion, we have isolated and characterized a new subfamily of ICK peptides from *M. charantia*. We characterized the CysI-CysIV, CysII-CysV, CysIII-CysVI disulfide connectivity and the cystine knot motif of MCh-1. The high yield of correctly folded MCh-1 *in vitro* and the accommodation of a wide range of sequences indicate it is a suitable framework for protein engineering applications. The intermediates isolated in the selective reduction and the oxidative refolding of MCh-1 were characterized, which indicated that this new family of peptides and other plant ICK peptides share a common, stable intermediate during folding.

## Supporting Information

File S1 Table S1 & S2, Figure S1 & S2. Table S1 Sequence fragments and sequences from the enzymatic digestion of the native and alkylated peptides from *M. charantia*. Figure S1 Quantification of Ia, IIa and MCh-1 observed during reductive unfolding and oxidative refolding in different time courses. (A) Reduced MCh-1 with 1 mM GSH. (B) Reduced MCh-1 without GSH. (C) IIa intermediate. Figure S2 TOCSY and NOESY spectra of MCh-1. (A) The TOCSY amide region and spin systems of MCh-1. (B) The NOESY fingerprint region and sequential connectivity via Hα-H_N_ of MCh-1. Table S2 NMR and refinement statistics of MCh-1 and MCh-2.
(DOC)
