# Supplementary material for: Novel Inhibitor Cystine Knot Peptides from Momordica charantia
Source: PLoS One. 2013 Oct 8;8(10):e75334. doi: 10.1371/journal.pone.0075334 (PMC3792974; doi:10.1371/journal.pone.0075334)
Supplement: File S1 — Table S1 & S2, Figure S1 & S2. Table S1 Sequence fragments and sequences from the enzymatic digestion of the native and alkylated peptides from M. charantia. Figure S1 Quantification of Ia, IIa and MCh-1 observed during reductive unfolding and oxidative refolding in different time courses. (A) Reduced MCh-1 with 1 mM GSH. (B) Reduced MCh-1 without GSH. (C) IIa intermediate. Figure S2 TOCSY and NOESY spectra of MCh-1. (A) The TOCSY amide region and spin systems of MCh-1. (B) The NOESY fingerprint region and sequential connectivity via Hα-HN of MCh-1. Table S2 NMR and refinement statistics of MCh-1 and MCh-2. (DOC) [file pone.0075334.s001.doc]

**Supporting Information**

# **Novel Inhibitor Cystine Knot Peptides from *Momordica charantia***

**Wen-Jun He (贺文军)^1,§^, Lai Yue Chan (陈丽如)^2,§^, Richard J. Clark^2,3^, Jun Tang (唐军)^1^, Guang-Zhi Zeng (曾广智)^1^, Octavio L. Franco^4^, Cinzia Cantacessi^5^, David J. Craik^2^, Norelle L. Daly^2,5,**^, and Ning-Hua Tan (谭宁华)^1,**^**

^1^State Key Laboratory of Phytochemistry and Plant Resources in West China, Kunming Institute of Botany, Chinese Academy of Sciences, Kunming, Yunnan, People’s Republic of China,

^2^Institute for Molecular Bioscience, The University of Queensland, Brisbane, Queensland, Australia,

^3^School of Biomedical Sciences, The University of Queensland, Brisbane, Queensland, Australia,

^4^Centro de Análises Proteômicas e Bioquímicas, Programa de Pós-Graduação em Ciências Genômicas e Biotecnologia, Universidade Católica de Brasília, Brasília-DF, Brazil,

^5^Centre for Biodiscovery and Molecular Development of Therapeutics, James Cook University, Cairns, Queensland, Australia

**Table S1**. Sequence fragments and sequences from the enzymatic digestion of the native and alkylated peptides from *M. charantia*.

|  | **Sequence Fragments and Sequences** | **Ions (Da)^a^** | **Experimental Mass (Da)** ^a^ | **Theoretical Mass (Da)^a^** | **Enzyme** |
| --- | --- | --- | --- | --- | --- |
| MCh-1 | GCAGKSCNILGSDPCDAGCF | 959.47^2+^ | 1916.94 | 1916.76 | C^b^ |
|  | --------------------CLPVGIVAGVCV | 565.26^2+^ | 1128.52 | 1128.61 | C |
|  | --------------------CLPVGIVAGVCV | 1129.51^+^ | 1128.51 | 1128.61 | C |
|  | -----SCNILGSDPCDAGCFCLPVGIVAGVCV | 1306.46^2+^ | 2610.92 | 2610.17 | T^c^ |
|  | GCAGKSCNILGSDPCDAGCFCLPVGIVAGVCV | 1512.00^2+^ | 3022.00 | 3021.35 |  |
| IAM-alkylated MCh-1 | GC(Am)AGKSC(Am)NILGSDPC(Am)DAGC(Am)F | 1073.49^2+^ | 2144.98 | 2145.00 | C |
|  | GC(Am)AGKSC(Am)NILGSDPC(Am)DAGC(Am)F | 715.99^3+^ | 2144.97 | 2145.00 | C |
|  | ------------------------------------C(Am)LPVGIVAGVC(Am)V | 622.37^2+^ | 1242.74 | 1242.73 | C |
|  | ---------SC(Am)NILGSDPC(Am)DAGC(Am)FC(Am)LPVGIVAGVC(Am)V | 1449.19^2+^ | 2896.38 | 2895.47 | T |
|  | ---------SC(Am)NILGSDPC(Am)DAGC(Am)FC(Am)LPVGIVAGVC(Am)V | 966.48^3+^ | 2896.44 | 2895.47 | T |
|  | GC(Am)AGKSC(Am)NILGSDPC(Am)DAGC(Am)FC(Am)LPVGIVAGVC(Am)V | 1686.13^2+^ | 3370.26 | 3369.65 |  |
| Ia (Oxidative refolding) | GC(NEM)AGKSC(NEM)NILGSDPCDAGC(NEM)F | 1146.84^2+^ | 2291.68 | 2292.14 | C |
|  | GC(NEM+H_2_O)AGKSC(NEM)NILGSDPCDAGC(NEM)F | 1155.86^2+^ | 2309.72 | 2310.14 | C |
|  | GC(NEM)AGKSC(NEM+H_2_O)NILGSDPCDAGC(NEM)F | 1155.86^2+^ | 2309.72 | 2310.14 | C |
|  | GC(NEM)AGKSC(NEM)NILGSDPCDAGC(NEM+H_2_O)F | 1155.86^2+^ | 2309.72 | 2310.14 | C |
|  | -----------------------------------C(NEM)LPVGIVAGVCV | 627.76^2+^ | 1253.52 | 1253.74 | C |
|  | -----------------------------------C(NEM)LPVGIVAGVCV | 1254.51^+^ | 1253.51 | 1253.74 | C |
|  | ----------SC(NEM)NILGSDPCDAGC(NEM)FC(NEM)LPVGIVAGVCV | 1493.97^2+^ | 2985.94 | 2985.55 | T |
|  | ----------SC(NEM)NILGSDPCDAGC(NEM)FC(NEM)LPVGIVAGVCV | 996.34^3+^ | 2986.02 | 2985.55 | T |
|  | GC(NEM)AGKSC(NEM)NILGSDPCDAGC(NEM)FC(NEM)LPVGIVAGVCV | 1763.58^2+^ | 3525.16 | 3525.85 |  |
| IIa (Oxidative refolding) | GC(NEM)AGKSCNILGSDPCDAGC(NEM)F | 1084.35^2+^ | 2166.70 | 2167.01 | C |
|  | GC(NEM+H_2_O)AGKSCNILGSDPCDAGC(NEM)F | 1093.35^2+^ | 2184.70 | 2185.01 | C |
|  | GC(NEM)AGKSCNILGSDPCDAGC(NEM+H_2_O)F | 1093.35^2+^ | 2184.70 | 2185.01 | C |
|  | ------------------------------CLPVGIVAGVCV | 565.26^2+^ | 1128.52 | 1128.61 | C |
|  | --------------------------------PVGIVAGVCV | 913.44^+^ | 912.44 | 912.52 | C |
|  | ------------------------------------VAGVCV | 547.23^+^ | 546.23 | 546.29 | C |
|  | ----------SCNILGSDPCDAGC(NEM)FCLPVGIVAGVCV | 1368.97^2+^ | 2735.94 | 2735.30 | T |
|  | ----------SCNILGSDPCDAGC(NEM)FCLPVGIVAGVCV | 912.77^3+^ | 2735.31 | 2735.30 | T |
|  | GC(NEM)AGKSCNILGSDPCDAGC(NEM)FCLPVGIVAGVCV | 1637.54^2+^ | 3273.08 | 3273.60 |  |
| IIa (Selective reduction) | GC(NEM)AGKSC(Am)NILGSDPC(Am)DAGC(NEM)F | 1141.32^2+^ | 2280.64 | 2281.13 | C |
|  | GC(NEM+H_2_O)AGKSC(Am)NILGSDPC(Am)DAGC(NEM)F | 1150.32^2+^ | 2298.64 | 2299.13 | C |
|  | GC(NEM+H_2_O)AGKSC(Am)NILGSDPC(Am)DAGC(NEM)F | 767.22^3+^ | 2298.66 | 2299.13 | C |
|  | GC(NEM)AGKSC(Am)NILGSDPC(Am)DAGC(NEM+H_2_O)F | 767.22^3+^ | 2298.66 | 2299.13 | C |
|  | ---------------------------------------------C(Am)LPVGIVAGVC(Am)V | 622.24^2+^ | 1242.48 | 1242.73 | C |
|  | ---------------------------------------------C(Am)LPVGIVAGVC(Am)V | 1243.54^+^ | 1242.54 | 1242.73 | C |
|  | -----------------SC(Am)NILGSDPC(Am)DAGC(NEM)FC(Am)LPVGIVAGVC(Am)V | 1483.06^2+^ | 2964.12 | 2963.54 | T |
|  | -----------------SC(Am)NILGSDPC(Am)DAGC(NEM)FC(Am)LPVGIVAGVC(Am)V | 989.07^3+^ | 2964.21 | 2963.54 | T |
|  | GC(NEM)AGKSC(Am)NILGSDPC(Am)DAGC(NEM)FC(Am)LPVGIVAGVC(Am)V | 1753.64^2+^ | 3505.28 | 3505.84 |  |
| MCh-2 | GCAGKACNLLGLTCDAGCF | 908.86^2+^ | 1815.72 | 1815.79 | C |
|  | -------------------CRPDGVGIVAGVCV | 672.85^2+^ | 1343.70 | 1343.68 | C |
|  | -----ACNLLGLTCDAGCFCRPDGVGIVAGVCV | 1363.57^2+^ | 2725.14 | 2725.26 | T |
|  | GCAGKACNLLGLTCDAGCFCRPDGVGIVAGVCV | 1568.64^2+^ | 3135.28 | 3135.45 |  |
| IAM-alkylated MCh-2 | GC(Am)AGKAC(Am)NLLGLTC(Am)DAGC(Am)F | 1022.97^2+^ | 2043.94 | 2044.03 | C |
|  | -----------------------------------C(Am)RPDGVGIVAGVC(Am)V | 729.90^2+^ | 1457.80 | 1457.80 | C |
|  | -----------------------------------C(Am)RPDGVGIVAGVC(Am)V | 1458.70^+^ | 1457.70 | 1457.80 | C |
|  | GC(Am)AGKAC(Am)NLLGLTC(Am)DAGC(Am)FC(Am)RPDGVGIVAGVC(Am)V | 3485.15^+^ | 3484.15 | 3483.75 |  |
| ^a^Monoisotopic masses are used;  ^b^Chymotrypsin digest;  ^c^Trypsin digest. | | | | | |

**Figure S1. Quantification of Ia, IIa and MCh-1 observed during reductive unfolding and oxidative refolding in different time courses.** (A) Reduced MCh-1 with 1mM GSH. (B) Reduced MCh-1 without GSH. (C) IIa intermediate.


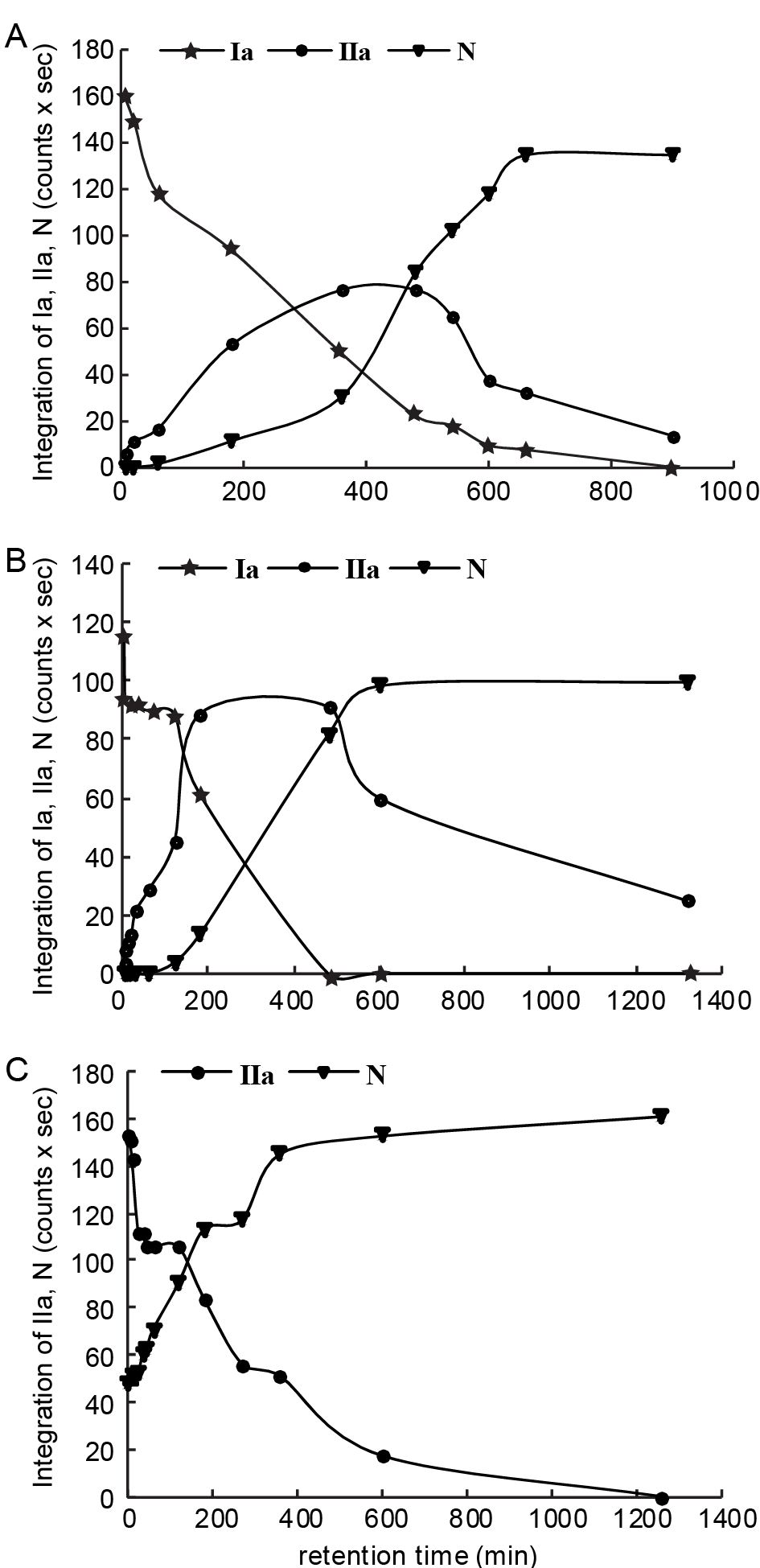


**Figure S2.** TOCSY and NOESY spectra of MCh-1. (A) The TOCSY amide region and spin systems of MCh-1. (B) The NOESY fingerprint region and sequential connectivity via Hα-HN of MCh-1.

**
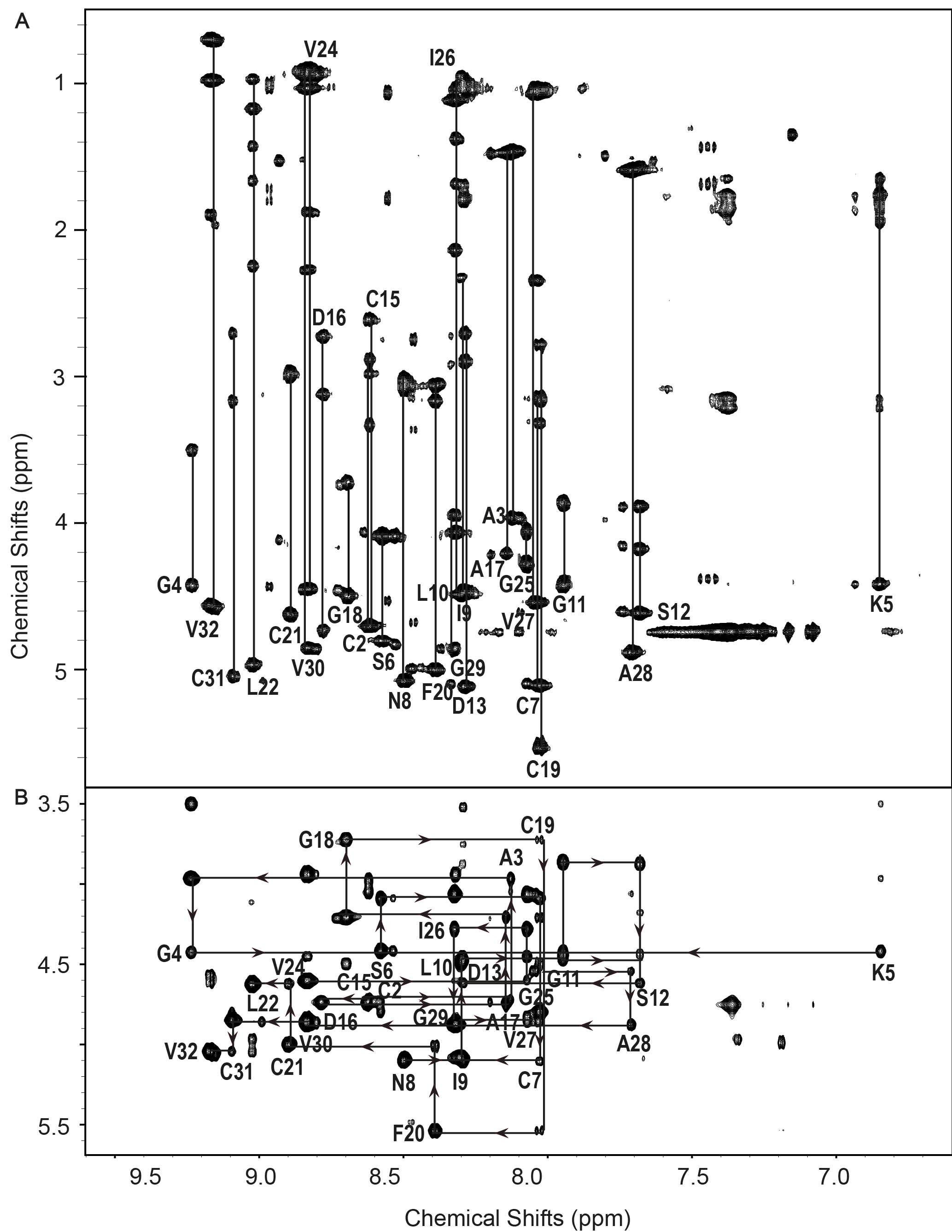
**

**Table S2**. NMR and refinement statistics of MCh-1 and MCh-2.

| **Pairwise RMSD (Å)** | **MCh-1** | **MCh-2** |
| --- | --- | --- |
| Mean global backbone | 0.54 ± 0.21 | 1.04 ± 0.33 |
| Mean global heavy atoms | 0.87 ± 0.27 | 1.57 ± 0.42 |
| Mean global backbone (residue 18-32)^a^ | 0.35 ± 0.14 | 0.31 ± 0.10 |
| Mean global heavy atoms (residue 18-32)^a^ | 0.60 ± 0.19 | 0.71 ± 0.18 |
| **Experimental data** |  |  |
| Distance restraints | 417 | 319 |
| Long range | 121 | 65 |
| Medium range | 61 | 52 |
| Sequential | 154 | 113 |
| Intra residues restraints | 81 | 89 |
| Dihedral restraints | 13 | 19 |
| NOE violations > 0.3 Å | 0 | 0 |
| Dihedral violations > 3.0° | 0 | 0 |
| **Energies (kJmol^-1^)** |  |  |
| Overall | -9.39E02 ± 0.87E01 | -1.03E03 ± 2.13E01 |
| Bonds | 0.63E01 ± 0.09E01 | 0.64E01 ± 0.11E01 |
| Angles | 2.79E01 ± 0.32E01 | 2.85E01 ± 0.46E01 |
| Improper | 0.42E01 ± 0.06E01 | 0.49E01 ± 0.13E01 |
| van der Waals | -5.48E01 ± 0.58E01 | -6.03E01 ± 0.56E01 |
| NOE | 1.67E01 ± 0.33E01 | 1.38E01 ± 0.77E01 |
| cDIH | 8.20E01 ± 0.02E01 | 0.06E02 ± 0.03E01 |
| Dihedral | 1.36E02 ± 0.75E01 | 1.15E02 ± 1.06E01 |
| Electrostatic | -1.08E03 ± 1.29E01 | -1.14E03 ± 2.27E01 |
| **RMSD^b^** |  |  |
| Bonds (Å) | 3.93E-03 ± 2.68E-04 | 3.86E-03 ± 3.13E-04 |
| Angles (°) | 4.96E-01 ± 2.82E-02 | 4.89E-01 ± 3.84E-02 |
| Improper (°) | 3.73E-01 ± 2.59E-02 | 3.89E-01 ± 4.82E-02 |
| NOE | 2.82E-02 ± 2.78E-03 | 3.39E-02 ± 7.92E-03 |
| cDIH | 3.91E-01 ± 3.19E-01 | 6.53E-01 ± 2.10E-01 |
| **Ramachandran (%)** |  |  |
| Most favoured | 85.2 | 81.7 |
| Additionally allowed | 14.6 | 13.7 |
| Generously allowed | 0.2 | 4.7 |
| Disallowed | 0 | 0 |
| All statistics are given as mean ± SD. |  |  |
| ^a^ Mean RMSD from MolMol. |  |  |
| ^b^ Average pairwise r.m.s.d. was calculated among 20 refined structures for mean global backbone and mean global heavy atoms. Structures were determined using CNS version 1.2 with the protocols outlined in Rosengren et al using the parameter and topology files, parallhdg5.2.pro and topallhdg5.2.pro respectively . | | |

1. Brünger AT, Adams PD, Rice LM (1997) New applications of simulated annealing in X-ray crystallography and solution NMR. Structure 5: 325-336.

2. Rosengren KJ, Daly NL, Plan MR, Waine C, Craik DJ (2003) Twists, knots, and rings in proteins–structural definition of the cyclotide framework. J Biol Chem 278: 8606-8616.

3. Linge JP, Habeck M, Rieping W, Nilges M (2003) ARIA: automated NOE assignment and NMR structure calculation. Bioinformatics 19: 315-316.
